# The effect of statins on sympathetic activity: a meta-analysis

**DOI:** 10.1007/s10286-015-0274-1

**Published:** 2015-03-05

**Authors:** Jacek Lewandowski, Bartosz Symonides, Zbigniew Gaciong, Maciej Siński

**Affiliations:** Department of Internal Medicine, Hypertension and Vascular Diseases, Medical University of Warsaw, Banacha 1a, 02-097 Warsaw, Poland

**Keywords:** Statin, Sympathetic nervous activity, Microneurography

## Abstract

**Objective:**

Beyond lipid-lowering properties, statins decrease sympathetic nervous activity. Due to the limited number of studies and included participants, a meta-analysis of randomized, placebo-controlled studies using microneurography (MSNA) was performed to assess sympatholytic effect of statins.

**Methods:**

We conducted a comprehensive search of online databases (Cochrane, Embase, and EBSCO) for published human studies up to April 2014. Randomized controlled trials (parallel and crossover design) were eligible for inclusion if results of statins versus placebo treatments on sympathetic activity were measured with MSNA.

**Results:**

Data from five studies with a total number of subjects *n* = 82 were included into the meta-analysis. MSNA expressed as bursts/min and as bursts/100 heartbeats was lower in the statin group than in the placebo group with a mean difference of −4.37 95 % CI (−7.03; −1.70), *p* < 0.0013 and −5.85 95 % CI (−7.56; −4.13), *p* < 0.0001, respectively. No significant publication bias was observed. Meta-regression revealed no significant effect of baseline total cholesterol or dose of statin. No change in blood pressure and heart rate was observed.

**Conclusions:**

Published data show that regardless of type and dose, statins reduce sympathetic activity measured by microneurography. The role of decreased sympathetic outflow during statin therapy on clinical end points needs to be clarified.

## Introduction

Statins are involved in numerous cholesterol-independent or pleiotropic effects [1, 2]. It is documented that statins interact with neurohumoral systems, particularly with the sympathetic nervous pathways [3]. Data from animal and human studies show that statins may decrease sympathetic nervous activity; however, the exact mechanism of that effect is not fully known [3]. Sympatholytic effects of statins were investigated in various populations using different methods of sympathetic drive analysis. Studies in which heart rate variability (HRV) was used as the indirect method of assessment of autonomic function showed conflicting results. After treatment with statins, increase in total power spectrum of HRV and reduction of low-/high-frequency spectra ratio (LF/HF) in frequency domain analysis as well as an increase in deviation of normal-to-normal intervals (SDNN) in time domain analysis was documented [4–6]. However, data showing no effects of statin therapy on both time and frequency domain indexes of HRV are also available [7, 8]. The results of studies with baroreflex control of heart rate (BRS) were more coherent and showed increase of BRS after statin treatment [9, 10]. The sympatholytic properties of statins were also examined using microneurography [11–18]. The number of studies as well as participating subjects was limited. In those studies, different populations of patients were examined using different compounds at different doses. Additionally, patients were either normo- or hypercholesterolemic.

Therefore, the aim of this study was to conduct a meta-analysis of randomized, placebo-controlled studies assessing the influence of statins on sympathetic activity measured with microneurography.

## Materials and methods

### Identification and selection of trials

We conducted a comprehensive search of online databases (Cochrane, PubMed, Embase and EBSCO) for published studies using a search strategy based on the words: statins or 3-hydroxy-3-methyl-glutaryl-CoA reductase inhibitors and microneurography and/or MSNA or muscle sympathetic nerve activity. The search was limited only to studies performed in humans and published up to April 10 2014. We also examined the reference lists of review articles and articles identified by electronic search to find any other eligible studies. The meta-analysis was prepared and tested in accordance with The PRISMA statement [19].

### Inclusion and exclusion criteria

The following criteria were applied for the studies to be included into the analysis:

1. Publication of original articles in peer-reviewed scientific journals.
2. Randomized controlled trial with comparison of statin with placebo.
3. Parallel or crossover design.
4. No statin use or washout period for statins before the study.
5. Stable treatment regimen before and during the study (with the exception of statin).
6. Information regarding post-treatment burst frequency (burst per minute) and/or burst incidence per 100 heartbeats assessed by MSNA.

Studies were excluded if they did not provide any information regarding the number of patients and post-treatment MSNA in the intervention and control groups. Two investigators performed the search independently and assessed the studies for eligibility. Disagreements were resolved by consensus. Database search revealed a total number of eight studies. Three of them were excluded because of no placebo group [16–18]. Therefore, five studies were included into the final analysis [11–15].

### Data extraction

Two investigators independently extracted the data from text, tables, and figures of eligible studies. We extracted publication year, sample size, age, gender, inclusion/exclusion criteria, name and dose of administered statin, duration of pretreatment phase with unchanged drug regimen, baseline blood pressure, heart rate, lipids, duration of treatment and pre- and post-treatment MSNA expressed as bursts/min and bursts/100 heartbeats.

### Statistical analysis

The meta-analysis was performed with R package (http://www.r-project.org, version 3.1.0) using “meta” package (version 3.5-1) and “metafor” package (version 1.9-3). A random effect model with inverse variance weighting for pooling and DerSimonian–Laird estimate was used. Bearing in mind the small number of studies, we refrained from testing the degree of heterogeneity between trials. We assessed the post-treatment between-group difference of MSNA for the parallel studies, and the difference between the post-statin and the post-placebo MSNA for crossover trials. Potential publication bias was assessed by visual inspection of a funnel plot and Egger’s test. The same methodology was applied for the assessment of post-treatment values of BP and HR. In all studies, clinic BP was used for analysis. In one study because of lack of post-treatment clinic BP, we used data from daytime ambulatory blood pressure monitoring [12].

Meta-regression was performed to assess whether the baseline total cholesterol and statin dose (expressed as equivalent dose of atorvastatin revealed by CURVES study) were associated with the effect of statin therapy on MSNA [20].

## Results

Five studies fulfilled the inclusion criteria. The characteristics of the investigations and the population studied are presented in Table 1. A total of 82 subjects with a predominance of males were included in the meta-analysis. In one study, only males were investigated [13]. The meta-analysis included subjects with a diagnosis of heart failure [11, 15] and essential hypertension [12–14]. Subjects with heart failure due to non-ischemic cardiomyopathy, were in the New York Heart Association (NYHA) class I–III with left ventricular ejection fraction (LVEF) assessed in echocardiography ≤35 or <40 % [11, 15]. Cholesterol levels were normal in two studies including subjects with heart failure, and in one study of subjects with hypertension [11, 14, 15]. In other studies, all subjects had hypercholesterolemia [12, 13].

**Table 1:** Baseline characteristics of included studies

References	*n*	Mean age (years)	Men (%)	Population studied	Statin (mg/day)	Treatment period (weeks)	SBP (mmHg)	DBP (mmHg)	HR (/min)	BMI (kg/m^2^)	T-chol (mg/dl)	MSNA (bursts/min)
Gomes [9]	13^a^	54	69.2	HT	A 80	3	152	92	71	26.7	208	39.2^b^
Lewandowski [10]	15/16	38.7	100	HT	S 40	8	142/136	91/86	77/75	28.7/27.1	249/232	36.5/33
Horwich [8]	9/9	48^c^	66.7^c^	HF	A 10	12	107/118	63/62	70/69	29/33	182/188	43/39
Deo [12]	6^a^	51	83.3	HF	S 40	4	119^b^	72^b^	74^b^	30	187^b^	44
McGowan [11]	14^a^	58	71.4	HT	S 80	4	139^b^	81^b^	59^b^	29.3	185^b^	32^b^

Data presented for total group or statin/placebo groups

*A* atorvastatin, *S* simvastatin, *HT* hypertension, *HF* heart failure

^a^Crossover design

^b^No data for the whole group in the crossover study. Data for post-placebo assessment

^c^Data for *n* = 26 patients, MSNA group was a substudy in *n* = 18 patients

The exclusion criteria of the studies were defined similarly and essentially excluded those with metabolic, endocrine, and neurological diseases or any other severe medical condition. In one study, active smokers or alcohol abusers were excluded [13]. Previous treatment of hypertension or heart failure was allowed, unless the treatment regimen was unchanged for at least 2 months before and during the survey [11–13]. Statins were not allowed for at least 2 months prior to the experiments. The time of investigational therapy ranged between 3 and 12 weeks. In all studies, only atorvastatin or simvastatin was used. A crossover design was used in three studies and direct comparison between two randomized groups in two studies.

**Fig. 1:**
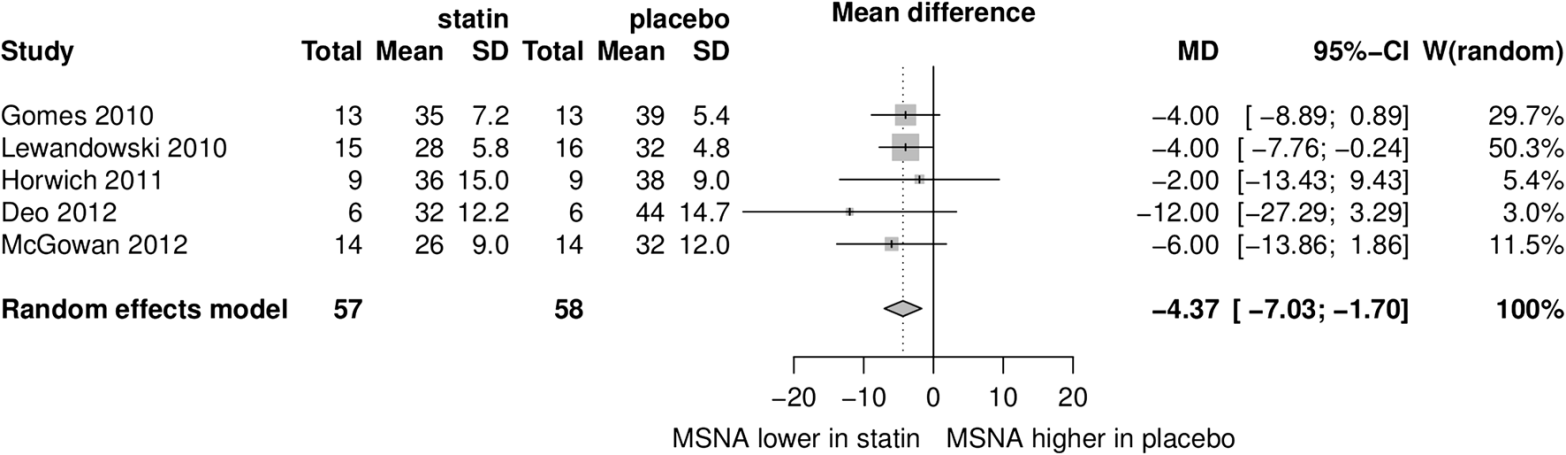
Mean differences in MSNA (bursts/min) between statins and placebo; *W* weighted

**Fig. 2:**
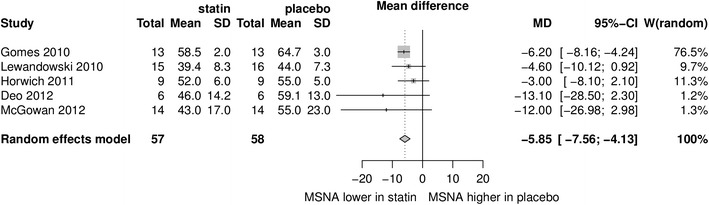
Mean differences in MSNA (bursts/100 heartbeats) between statins and placebo; *W* weighted

**Fig. 3:**
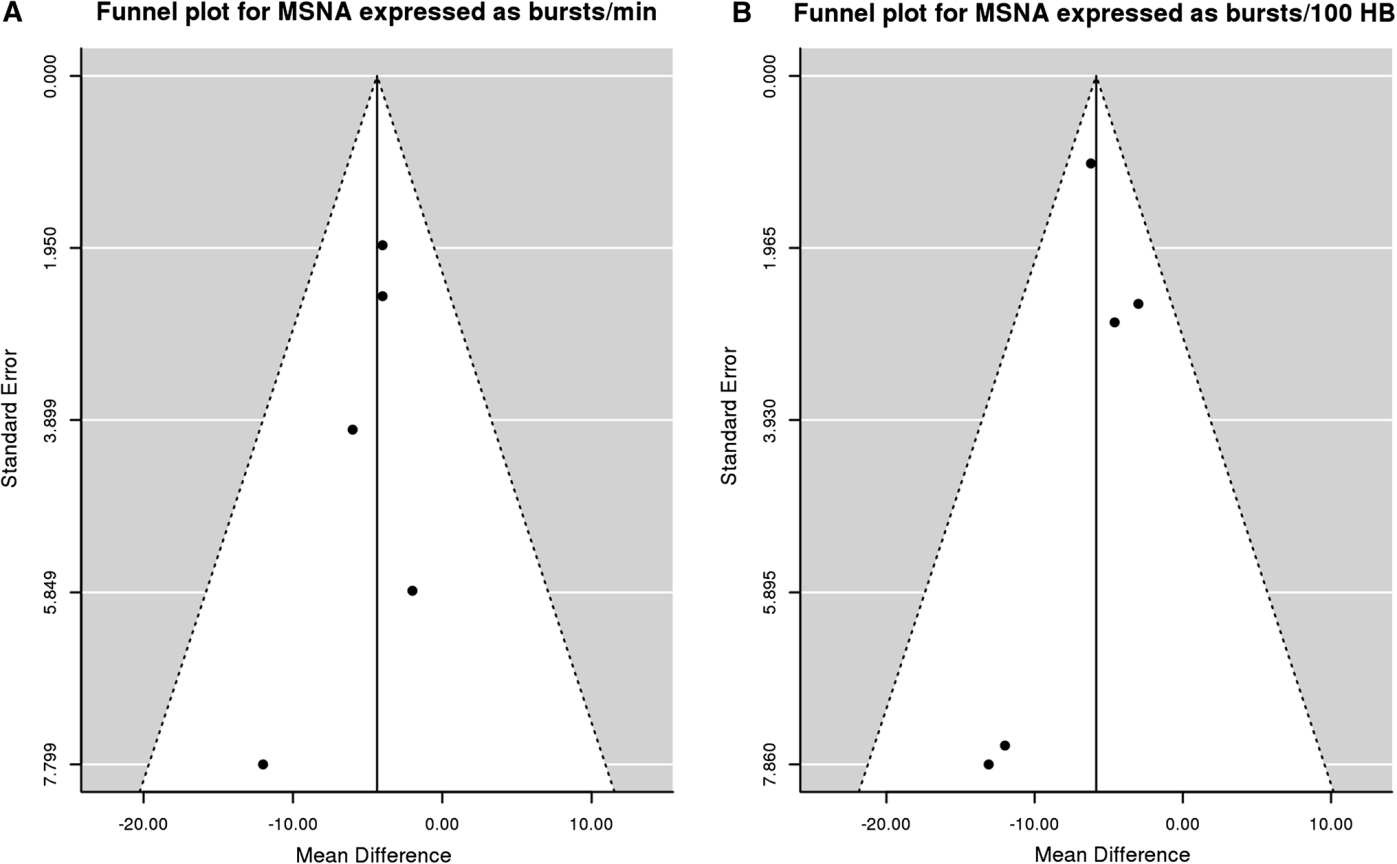
Funnel plot for MSNA expressed as bursts/min (**a**) and bursts/100 heartbeats (**b**)

**Fig. 4:**
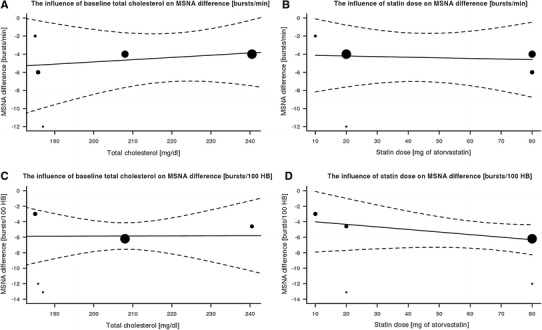
Meta-regression: the influence of baseline total cholesterol (**a**, **c**) or dose of statin (equivalent for atorvastatin) (**b**, **d**), for MSNA difference expressed as bursts/min (**a**, **c**) or bursts/100 heartbeats (**b**, **d**)

MSNA expressed as burst/min was lower in the statin group than in the placebo group with a mean difference of −4.37 95 % CI (−7.03; −1.70), *p* < 0.0013. MSNA expressed as burst/100 heartbeats was also lower in the statin group than in the placebo group with a mean difference −5.85 95 % CI (−7.56; −4.13), *p* < 0.0001. The results are presented in Figs. 1 and 2. No significant publication bias for MSNA/min and MSNA/100 heartbeats was revealed by visual inspection of the respective funnel plots (Fig. 3) and Egger’s test (z = −0.65, *p* = 0.52 and z = −0.72, *p* = 0.50 respectively). Meta-regression revealed no significant effect of baseline total cholesterol or dose of statin on MSNA changes (Fig. 4). Systolic blood pressure (SBP) was similar in the statin and placebo group −1.66 95 % CI (−7.26; 3.94), *p* = 0.60 as well as diastolic blood pressure (DBP) −0.84 95 % CI (−4.47; 2.80), *p* = 0.65 and heart rate (HR) −0.65 95 % CI (−4.01; 2.71), *p* = 0.70.

## Discussion

The main finding of the present meta-analysis is that statins, as compared to placebo, reduce sympathetic activity as measured by microneurography. To clarify the effects of statins on sympathetic outflow, we selected only those studies where microneurography was performed. Microneurography is a direct method to measure sympathetic outflow, the results of which highly correlate with other methods of sympathetic activity investigation, including norepinephrine release [21]. In online databases, we found five studies only five studies evaluating the effect of statins on the sympathetic activity which met the inclusion criteria, including the use of microneurography and placebo as a comparator. In all studies, a limited number of participants were included, with a significant predominance of males. So far, no convincing data are available to prove that statins may exert different lipid-lowering effects or clinical outcomes depending on sex. In currently analyzed studies, participants with both normal and high cholesterol levels were included. Although the central mechanisms of sympathoinhibitory effect of statins is suggested, the relationship between lipid-lowering effects of drugs and their sympathoinhibitory effect remains unclear [22]. The results of the presented meta-analysis confirm findings from non-placebo-controlled studies [16–18]. In one study 6 weeks therapy with atorvastatin in patients with chronic kidney diseases resulted in decrease of MSNA, while BP was unchanged [16]. In our study, 8 weeks therapy with atorvastatin decreased MSNA and increased BRS, but BP was unchanged [17]. In another study of patients with heart failure, therapy restarted with statin also decreased MSNA, while it did not affect plasma norepinephrine or BP [18].

The studies included in the meta-analysis involved subjects with normal blood pressure, arterial hypertension, and heart failure. Only one study, involving patients with non-ischemic heart failure (HF), failed to demonstrate a substantial decrease of sympathetic activity after statins [11]. The authors noted that the negative results of the study might depend on the low dose of atorvastatin (10 mg) and the relatively low severity of heart failure, which was translated into less pronounced neurohumoral excitation. Moreover, in all subjects, optimal therapy was continued, including beta adrenolytics, ACE inhibitors/sartans and aldosterone antagonists. These could have diminished the potential effect of statins on sympathetic activation. One may speculate that the use of cardiovascular drugs may influence the effects of statins, but accompanying therapy was started before the studies and remained unchanged during the experimental period. Moreover, in several studies, the effects of cardiovascular drugs on sympathetic activity have been ambiguous—even between various preparations within the same drug class. Therefore, the changes detected in sympathetic drive might be solely attributed to the effects of statins. In daily practice, however, it should be underlined that modification of sympathetic drive might not only be the effect of statins but also the effect of concomitant treatment.

The new finding of the current meta-analysis is that sympathoinhibitory effect of statins was not related to the dose of statin, since meta-regression revealed no significant relationship between the dose of the drug and post-treatment MSNA difference. This observation may indirectly support the hypothesis that the sympathoinhibitory effect of statin is cholesterol independent and therefore results from pleiotropic effects of statins. It should be noted, however, that in the analyzed studies only the effects of simvastatin and atorvastatin were investigated. Both of these statins are lipophilic and have a greater potential to cross the blood–brain barrier. Consequently, they have a greater potential to influence the central nervous system regions involved in the modulation of autonomic balance. Up-to-date studies on sympathetic activity with hydrophilic statins such as pravastatin or rosuvastatin are limited and so far the studies in humans with the use of microneurography are lacking. Hence, the current conclusion concerning the sympatholytic effects of statins should be cautiously stated and relate only to simvastatin and atorvastatin.

Meta-regression also revealed no significant relationship between the baseline cholesterol and post-treatment MSNA difference, which further and more directly supports the lipid independent effects of statins on sympathetic activity, as suggested by other authors [3]. Recently we showed that in hypertensive patients with hyperlipidemia, simvastatin and ezetimibe exerted similar hypolipidemic effects, although only the statin reduced sympathetic activity [23]. It may strongly support the idea that the sympatholytic effect of statins is independent of changes in plasma cholesterol.

The presented meta-analysis showed no influence of statins on BP or HR changes, however, that may be due to the limited number of subjects enrolled in the analysis. Earlier, this issue was addressed in some other meta-analyses, which showed an ambiguous effect of statins on BP [24, 25].

The available evidence indicates that there is a strong positive correlation between sympathetic activity and insulin resistance [3]. Therefore, it might be confusing that statins exert simultaneously both sympatholytic and prodiabetogenic effects. Interestingly, in one study included in the meta-analysis, simvastatin reduced sympathetic activity without improvement in insulin resistance [14]. Recently, it was documented that statins might be involved in an inhibition of glucose uptake, which may be one of the putative mechanisms explaining the diabetogenic effect of statins [26].

A few limitations of the current meta-analysis should be mentioned. We purposely refrained from testing the degree of heterogeneity between trials and selected the random effect model. The tests for estimation of the heterogeneity in a small meta-analysis which includes only few trials usually provide an incorrect zero between study variance estimates, leading to a false homogeneity assumption. Heterogeneity is consistently underestimated in meta-analyses [27]. Some authors express an opinion that there is no infallible method to test whether the true effects are really homogeneous or not, and that a researcher should decide on the type of inference desired before examining the data and choosing the model accordingly [28]. In our meta-analysis, the selection of the random effect model was, according to our opinion, more appropriate than the fixed effect model, since the studies differed regarding the underlying disease, baseline lipid, statin formulation, and duration of treatment. Therefore, the expected effect size would be similar, but not identical across studies, making the use of the random effect model more appropriate.

The practical implications of the presented findings might be highly relevant for the management of cardiovascular diseases. Statins have been commonly used in the therapy of cardiovascular disorders owing to their ability to lower total cholesterol and LDL cholesterol. Later evidence extends the use of statins beyond their lipid-lowering capabilities to a broader patient population. There is currently no consistent evidence that pleiotropic effects of statins, including sympathoinhibitory properties, translate into a long-term reduction of cardiovascular episodes. Nevertheless, in the last European guidelines for the managements of dyslipidemias, plasma LDL targets have been further reduced in respective groups of patients to decrease cardiovascular disability and death rates [29]. Additionally, the recent American guidelines make no recommendations for specific LDL-cholesterol targets for both the primary and secondary prevention of cardiovascular diseases [30]. However, certain patients were identified, in whom statin therapy should be used to reduce cardiovascular events rather than to achieve specified LDL goals. Thus, although lipid-lowering properties remain the primary measure of statins clinical efficacy, their numerous pleiotropic activities might be also taken under consideration in the prevention of cardiovascular diseases.

Since sympathetic hyperactivity is implicated in the pathogenesis of diseases, the use of HMG-CoA reductase inhibitors as a sympathoinhibitory agent is highly attractive. Because the sympatholytic effect of statins seems to be independent of their lipid-reducing properties, the use of these drugs may also be justified to include cardiovascular patients even with normal lipid levels.
